# Single domain antibodies from camelids in the treatment of microbial infections

**DOI:** 10.3389/fimmu.2024.1334829

**Published:** 2024-05-17

**Authors:** Henri De Greve, Antonella Fioravanti

**Affiliations:** ^1^ Structural Biology Brussels, Vrije Universiteit Brussel, Brussels, Belgium; ^2^ VIB-VUB Center for Structural Biology, Vrije Universiteit Brussel, Brussels, Belgium; ^3^ Fondazione ParSeC – Parco delle Scienze e della Cultura, Prato, Italy

**Keywords:** nanobodies (Nbs), infectious diseases, novel therapy for infectious diseases, passive immune therapy, antiviral therapies, antimicrobial therapy, antimicrobial resistance (AMR), antibiotic resistance

## Abstract

Infectious diseases continue to pose significant global health challenges. In addition to the enduring burdens of ailments like malaria and HIV, the emergence of nosocomial outbreaks driven by antibiotic-resistant pathogens underscores the ongoing threats. Furthermore, recent infectious disease crises, exemplified by the Ebola and SARS-CoV-2 outbreaks, have intensified the pursuit of more effective and efficient diagnostic and therapeutic solutions. Among the promising options, antibodies have garnered significant attention due to their favorable structural characteristics and versatile applications. Notably, nanobodies (Nbs), the smallest functional single-domain antibodies of heavy-chain only antibodies produced by camelids, exhibit remarkable capabilities in stable antigen binding. They offer unique advantages such as ease of expression and modification and enhanced stability, as well as improved hydrophilicity compared to conventional antibody fragments (antigen-binding fragments (Fab) or single-chain variable fragments (scFv)) that can aggregate due to their low solubility. Nanobodies directly target antigen epitopes or can be engineered into multivalent Nbs and Nb-fusion proteins, expanding their therapeutic potential. This review is dedicated to charting the progress in Nb research, particularly those derived from camelids, and highlighting their diverse applications in treating infectious diseases, spanning both human and animal contexts.

## 1 Introduction

**Figure 1:**
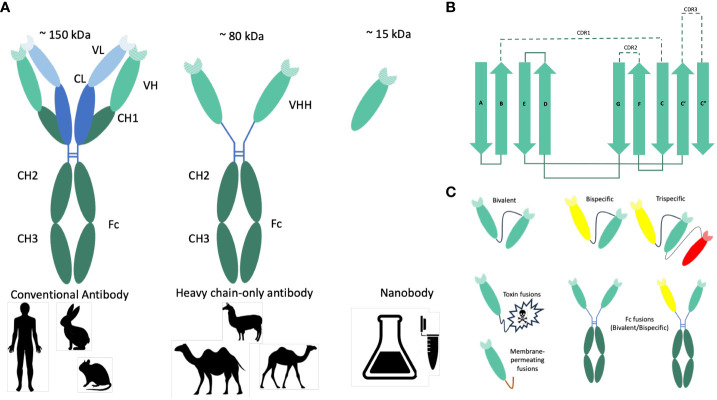
Origin, structure and formats of nanobodies. **(A)** Graphical representation of conventional and heavy-chain-only antibodies. Conventional antibodies are found in mammals. They consist of two heavy (green) and two light (blue) chains, and their antigen-binding region (paratope) is encoded by the variable domains of both chains (VH and VL). In camelids (camels, dromedary, Ilama, and alpaca), next to the conventional antibodies, heavy-chain-only antibodies are also found. The antigen is recognized by the variable domain of the heavy chain (VHH). Nanobodies (Nbs) are VHHs derived from heavy-chain-only antibodies with a size of approximately 15 kDa. **(B)** Schematic representations of the nanobody architecture. Nanobodies comprise four framework regions (FR1–4) and three hypervariable regions (CDR1–3). The structural architecture of nanobodies includes 2 β-sheets, one with 4 β-strands (A, B, D, and E) and one with one β-strand (C, C’, C”, F, and G). CDR1, CDR2, and CDR3 are unstructured loops. **(C)** Nanobody-based engineered molecules to improve the antimicrobial potency or stability of anti-pathogenic Nbs. Due to their modular structure, Nbs can function as building blocks in multimeric constructs binding the same (multivalent) or different (multiparatopic) epitopes. Monovalent Nbs can be conjugated genetically to toxins to promote target-cytotoxicity or to membrane-permeating peptides to allow entry into target cells. To produce bivalent or bispecific recombinant antibodies and to mediate different effector functions, Nbs can be fused to the constant Fc domain of conventional IgG or IgA antibodies. Monomeric, dimeric (bivalent or biparatopic) or trimeric (trivalent or trispecific) Nbs can be obtained by linking the different monovalent Nbs head to tail using a linker peptide.

More than 125 years ago, Behring and Kitasato (1) showed that hyperimmune sera from animals immunized with inactivated *Corynebacterium diphtheriae* or *Clostridium tetani* could protect treated animals from disease caused by the same virulent pathogenic bacteria. This passive immunotherapy was rapidly adapted to treat diphtheria outbreaks in humans with dairy cow-derived polyclonal diphtheria-immune serum (2). Today, this approach is still in use, employing commercial antisera produced by humans or animals to combat a wide range of toxins, bacteria and viruses (3). Advancements in technology, including the groundbreaking discovery of monoclonal antibodies (mAbs), the application of diverse methods for screening large antibody libraries (4), together with recombinant DNA technologies, have paved the way for the development of chimeric mAbs. These antibodies replace the native murine heavy chain constant region with its human counterpart (4). Heavy-chain-only antibodies were discovered in the early 1990s within the Hamers’ laboratory at Vrije Universiteit Brussel (5). These antibodies consist of two heavy chains and are only found in members of the Camelidae family, such as llamas and camels. Sharks also employ distinct mechanisms to produce single-domain antibodies (6). The camelid heavy-chain-only antibodies (HCabs) differ from the typical antibody structure by lacking the variable and constant light chain (VL-CL) and the constant domain (CH1) (**Figure 1A**). Instead of the classical variable heavy chain domain (VH), they consist of a single variable heavy chain domain (VHH) that is linked by a flexible hinge to the Fc domain (CH2-CH3) and is responsible for antigen binding. HCabs are generated by VDJ recombination, where the variable region is VHH rather than classical VH germline sequence, followed by somatic hypermutation upon immunization with the specific antigen/s. VHHs or nanobodies (Nbs) are characterized by their diminutive size of approximately 12–15 kDa. They are built from four framework regions (FR1, FR2, FR3 and FR4) and three hypervariable complementarity-determining regions (CDR1, CDR2 and CDR3) (7, 8) (**Figure 1B**). To enhance the solubility of VHHs, hydrophobic amino acids in frame 2 (FR2) of the germline were replaced with hydrophilic ones. Namely, the four hydrophobic amino acids in the FR2 (V42, G49, L50 and W52), which typically mediate interdomain interactions between conventional VH and VL domains, are substituted by hydrophilic amino acids (F42, E49, R50 and G52) (9).

A distinct hallmark of Nbs is the presence of a long protruding loop within the third complementarity-determining region (CDR3), which plays a pivotal role in antigen recognition and binding. It is this highly variable CDR3 region that makes a significant contribution to antibody diversity and specificity. This peculiar conformation of CDR3 allows nanobodies the ability to access concealed or cryptic epitopes, a feat often beyond the reach of traditional antibodies (10, 11) (**Figure 1A**).

In this review, only Camelidae-derived Nbs and their therapeutic application will be discussed. Monomeric Nbs are easily obtained by standardized procedures for their identification, production and purification (9, 12, 13). They also present higher stability to pH and temperature extremes than conventional antibodies (9, 14). While these Nbs exhibit remarkable potential for therapeutic applications, their short circulating half-life presents a significant challenge (15). However, ongoing research has identified several strategies to mitigate this limitation, including fusion with the Fc region of immunoglobulin G (IgG) or immunoglobulin A (IgA), PEGylation, glycosylation, and albumin binding (16, 17). Monovalent Nbs’ half-life can be prolonged by linking them to an albumin-specific Nb, thereby diminishing the renal filtration-induced loss of Nbs (18). Alternatively, fusing Nbs to the Fc domain of IgG increases the monomeric Nb half-life from 30 min up to 7 to 10 days for the Nb-IgG chimeric antibody (19). The Fc regions of IgG and IgA activate the immune system through binding to Fc receptors (20).

Fusing the Fc domain of IgG is the favored choice for systemic intravenous application of Nbs, while the Fc domain of IgA would be preferred for their mucosal application. Furthermore, chimeric secretory IgA (sIgA) fused to Nbs can be produced cost-efficient in plants (21, 22). These approaches aim to enhance nanobodies’ pharmacokinetic properties and tissue distribution, thereby maximizing their therapeutic efficacy. Injected monomeric (humanized) Nbs present a low immunogenicity risk profile (9, 23). Therefore, it can be assumed that the fusion of Nbs to Fc domains of the target animal or human will not generate an immune response in the circulation. We could also hypothesize that the mucosal immune response against Nbs (multivalent or to Fc domains) would be none or very low.

Despite the challenges, nanobodies offer promising avenues for combating infectious diseases, particularly microbial infections. Their high specificity, affinity, and ability to target conserved epitopes, some of which are often encrypted and inaccessible to larger antibody formats, make them attractive candidates for microbial and antiviral therapies. Successful translation of nanobody-based therapies into clinical applications hinges on addressing key considerations such as target specificity, tissue distribution and modulatory effects. As research progresses, nanobodies hold immense potential to revolutionize the landscape of infectious disease treatment, offering novel strategies to combat emerging pathogens and improve patient outcomes.

In this review, we will focus on the promising application of Nbs for passive immunization in preventing and treating bacterial and viral infections. Specifically, we will explore their development and potential utilization in select examples of bacterial and viral diseases. The utilization of monomeric or dimeric Nbs, which can bind multiple epitopes, in addressing bacterial and viral infections will also be discussed. Furthermore, we will explore the potential benefits of fusing Nbs with Fc domains of IgAs or IgGs to combat infectious disease in both human and animal contexts, considering their potential advantages as Fc fragments dimerize the molecule and enable their interaction with the Fc receptors on the host cell surface (24).

## 2 Nanobodies against pathogenic bacteria

### 2.1 Nanobodies to treat pathogenic *Escherichia coli* infections

#### 2.1.1 Post-weaning diarrhea in piglets

Post-weaning diarrhea (PWD) is a common issue in piglets and it is predominantly caused by enterotoxigenic *E. coli* strains (ETEC) (25). The initial three weeks post-weaning represent a critical period for piglet vulnerability to ETEC infection. Beyond this timeframe, pigs are less vulnerable to ETEC infection and will exhibit heightened resistance, mounting an immune response to ETEC. PWD is characterized by manifestations like diarrhea, dehydration, stunted growth, and mortality (25). Stressors such as the separation from the sow, transition from maternal milk to solid feed, relocation to new facilities, and mixing with piglets from different sources amplify piglet susceptibility to ETEC infections. ETEC strains causing PWD produce enterotoxins accountable for the symptoms and typically employ F4 or F18 fimbrial adhesins, which facilitate colonization within the small intestine (26).

In the past, antibiotics were commonly administered to piglets to prevent PWD. This was due to the fact that attempts to obtain active immunization, aiming to induce a mucosal immune response against the F4 or F18 colonization factors, faced interference from anti-F4 or anti-F18 antibodies present in the sow’s milk. Consequently, antibiotics like colistin were incorporated into the diet of weaning piglets. However, in 2006, the European Union prohibited the prophylactic use of antibiotics in animal feed. This decision, reflected in regulations such as 1831/2003/EC on additives for animal nutrition, was implemented to curtail the emergence of antibiotic resistance in humans.

Since generating an active mucosal immune response against the F4 or F18 fimbriae proved challenging, Virdi and colleagues tested whether oral feed-based passive immunization by adding recombinant anti-F4 Nbs fused to the Fc domains of porcine IgG or IgA (**Figure 1C**) could prolong the maternal lactogenic immunity during the first three weeks and prevent PWD in weaned piglets (21). All extracts of the recombinant anti-F4 Nb-IgG and Nb-IgA produced in plant seeds showed *in vitro* inhibition of bacterial binding to porcine gut villous enterocytes. In a challenge experiment, only the secretory IgA-like recombinant anti-F4 Nb-IgA showed *in vivo* protection of the piglets against colonization by F4-positive ETEC (21). Later, it was demonstrated that also the monomeric form of the recombinant anti-F4 Nb-IgA produced in yeast, Arabidopsis and soybean was as efficient as the secretory IgA-like recombinant anti-F4 Nb-IgA (27).

In literature, it has been demonstrated that linking two or more neutralizing Nbs recognizing different epitopes into hetero-multimers often significantly improves their *in vivo* neutralizing potency (28–30).

Thus, an alternative strategy utilizing Nbs to prevent PWD involves the addition of purified bivalent monomeric Nbs to the feed of weaned piglets. Recently, Fiil and colleagues demonstrated that a bivalent monomeric anti-F4 Nb inhibits the *in vitro* binding of F4-positive bacteria to pig enterocytes (31). Furthermore, this Nb reduces the *in vivo* colonization time of weaned piglets when exposed to an F4-positive ETEC strain. However, in terms of weight gain, diarrhea severity, and essential blood response parameters, such as hematocrit value and leukocyte counts, no significant variations were detected between the challenged piglets receiving the bivalent monomeric anti-F4 Nb and the control group. This contrasts with the findings of Virdi et al. (2013), who observed significant differences between challenged control or anti-F4 Nb-IgG piglets and the piglets receiving the anti-F4 Nb-IgA. In the latter study, reduced shedding of the F4-positive ETEC strain and diminished immune responses corroborated a reduced exposure to the ETEC pathogen and a significantly higher weight gain (21). These varying outcomes in the studies may be attributed to the presence or absence of the Fc domain of the pig IgA, with the IgA Fc domain possibly enhancing effector functions and mucosal stability in the intestine.

#### 2.1.2 Diarrhea in young children and travelers

In developing countries, enterotoxigenic *E. coli* (ETEC) strains that produce colonizing factor (CF) or coli surface (CS) antigens are a common cause of diarrhea in children under the age of five, a condition that can unfortunately lead to fatal outcomes. These ETEC strains also affect adults within these regions and individuals traveling to endemic areas, giving rise to symptoms like watery diarrhea combined with vomiting, stomach cramps, and mild fever (32).

Developing vaccines that actively protect against the human ETEC strains remains challenging because more than 25 types of CF or CS antigens have been identified (33–35). Not too long ago, a cross-protective vaccine that contains a combination of four intact CF antigens, ETVAX, was developed (36). This multivalent vaccine provided good protection in Bangladeshi children (37, 38) but showed poor or no protection in Egyptian children (39). Although no licensed vaccine against human ETEC strains is currently on the market, a commercial product called Travelan has been developed (Immuron, Australia). Travelan is a gluten-free bovine colostrum plus lactose that can be obtained over the counter in the USA, Australia and Canada. It contains anti-ETEC antibodies and is currently used to prevent traveler’s diarrhea, but it does not provide efficient protection. To overcome this high antigenic diversity of the fimbrial antigens, llama Nbs that recognize the minor fimbrial adhesin CfaE were generated. These Nbs cross-reacted with 11 pathogenic ETEC strains and prevented *in vivo* colonization of mice when challenged by five out of six different ETEC strains (40). Moreover, one of these Nbs was fused to the Fc domain of IgA1 (**Figure 1C**), and oral administration of this fusion protein showed a prolonged inhibitory activity in mouse colonization at a lower dose than Travelan (40). Since several Nbs with a broad host range were isolated against the fimbrial adhesin CfaE (40), it is possible that a combination of two or more Nbs fused to the Fc domain of IgA1 could further enhance the protection against ETEC strains.

#### 2.1.3 Enterohemorrhagic *E. coli* infections

Enterohemorrhagic *E. coli* (EHEC) strains cause life-threatening infections in developed countries. EHEC infections can lead to hemorrhagic colitis and hemolytic uremic syndrome (HUS), injuring the kidneys (41, 42). The main sources of human EHEC infections are undercooked meat from cattle or vegetables cross-contaminated by manure from cattle (43). Upon infection, EHEC injects the translocated intimin receptor (Tir) into the intestinal epithelial cells via a type III secretion system (44, 45). Tir becomes integrated into the host cell membrane, and its central region (TirM) forms a 109-amino acid loop that protrudes outside the cell (46, 47). This loop binds to the C-terminal domain of intimin (48–50), resulting in a close interaction between the bacterial cell and the host cell, leading to the formation of a pedestal-like structure (44, 51, 52). The Nb TD4, obtained from immunized dromedary, recognizes and binds to TirM with an affinity of 4.8 nM, which is 10-fold higher than the affinity of intimin (53, 54). TD4 binds to HeLa cells presenting Tir and inhibits or significantly reduces the number of EHEC bacteria attached to HeLa cells. Similarly, four Nbs were obtained against the C-terminal 277 residues of intimin (Int-277) of EHEC O157:H7 strain EDL933. These Nbs were fused to bovine IgA and produced as chimeric secretory sIgA in leaves of *Nicotiana benthamiana* (22). These plant-produced chimeric secretory Nb-sIgAs were purified and bind specifically to the intimin antigen. Additionally, one of the plant-produced VHH10-sIgA that binds to the seven most prevalent EHEC strains (serotypes O111:Hnm, O26:H11 and O157:H7) also reduces *in vitro* the adherence of EHEC strains to epithelial cells (22).

The anti-intimin or anti-tir Nbs open possibilities for passive immunization and therapeutic strategies to prevent EHEC adhesion to intestinal tissues during human infection. Additionally, these Nbs could also be used to reduce both the prevalence of EHEC in cattle and decrease EHEC contamination of the food chain.

#### 2.1.4 Shiga toxins

Shiga toxins are responsible for systemic disease symptoms such as hemorrhagic colitis and hemolytic uremic syndrome (HUS) in humans (41, 42, 45) and edema disease in piglets (55). Two main classes of Shiga toxins, Stx1 and Stx2, are made by Shiga toxin-producing *E. coli* strains (56). Stx1 differs in only one amino acid from the Shiga toxin Stx produced by *Shigella dysenteriae* strains. Stx2 is antigenically different from Stx1 and differs by more than fifty percent from Stx1. Shiga toxins are built up of an enzymatically active A subunit and five B subunits that bind to a specific glycolipid receptor on host cells. All Shiga toxins, except Stx2e, preferentially bind to the glycolipid receptor globotriaosyl ceramide Gb3 present on the surface of epithelial cells in the intestine and endothelial cells in the kidney (57–60). The Stx2e toxin, on the other hand, predominantly binds to the glycolipid receptor globotetraosyl ceramide Gb4 (61). The Shiga toxins cross the epithelial barrier of the colon and are transported via the bloodstream to the target organs, such as the kidneys, carrying a high number of Gb3 receptors.

Given that severe illness results from the systemic action of the Shiga toxins, an alternative therapeutic approach to prevent or treat the main symptoms caused by these toxins is to neutralize the Shiga toxin in the blood with anti-Stx Nbs. Several research groups have successfully isolated neutralizing anti-Stx2 Nbs (62–64). They used a recombinant BLS-Stx2B fusion protein between the *Brucella* lumazine synthase (BLS) and the B subunit of Stx2a (65) to immunize llama and to isolate neutralizing Nbs against the Stx2B subunit (63). One of these *in vitro* neutralizing anti-Stx2B Nb (2vb27) was dimerized and fused to an anti-human seroalbumin (SA) Nb, increasing the Nb’s half-life in circulation. Additionally, a trimeric construct, (2vb27)_2_-SA, demonstrated the ability to neutralize Stx2 *in vivo* in various mouse models (63), showing the potential to heal HUS in humans. In another study, Nbs were generated in an alpaca with only the recombinant Stx2a B subunit (rStx2aB) as antigen (64). Among the isolates Nbs, Nb113 showed the highest affinity against rStx2aB and was used to study the molecular interaction with the rStx2aB pentamer by X-ray crystallography. The study revealed that a single Nb113 binds to each Stx2aB subunit in the pentamer, effectively concealing the Gb3 receptor-binding sites present on the B subunits. *In vitro* experiments on Vero cells using the monovalent Nb113 (a bivalent (Nb113)_2_ construct) and a trimeric (Nb113)_2_–NbSA1 construct (which combined a bivalent (Nb113)_2_ with an Nb against serum albumin), showed that the bivalent and trimeric constructs led to increase *in vitro* neutralization of the Stx2a toxin (64) (**Figure 1C**).

Next to humans, pigs are very sensitive to the Stx2e variant of the Stx2 toxin family, causing edema disease in weaned piglets. The Stx2e-producing *E. coli* strains express F18 fimbriae on the bacterial surface, mediating colonization of the intestine of the piglets (55). Piglets can be passively protected against edema by vaccination of pregnant sows with a Stx2e toxoid (66). This Stx2e toxoid was used to obtain 8 Nbs against the Stx2e toxoid (62). One of these Nbs, NbStx2e1, showed a potent neutralizing capacity against the Stx2e toxin in a Vero cell assay. The crystal structure of the complex between NbStx2e1 and the Stx2e toxoid showed that one NbStx2e1 interacts in a head-to-head orientation with each B subunit of Stx2e. The binding of the NbStx2e1 to the B subunits directly competes with the glycolipid receptor binding site on the surface of the B subunit (62). NbStx2e1, with its potent neutralization capacity, represents a promising candidate for the prevention or treatment of edema disease in weaned piglets.

### 2.2 Nanobodies neutralizing *Listeria monocytogenes*

*L. monocytogenes* is a food-borne disease that causes severe gastroenteritis and can also lead to fatal meningitis (67). Pregnant women are particularly vulnerable to *L. monocytogenes* infections (68).

The initial stage in the infection process of *L. monocytogenes* involves the invasion of host cells, which occurs through receptor-mediated endocytosis (69). The entry of the host cell is directed by two virulence factors members of the internalin family, InlA and InlB (70, 71). InlA binds to E-cadherin present on the surface of intestinal epithelial cells (72), while InlB binds to the tyrosine kinase c-Met receptor present on HeLa and Vero cells (73). Once inside the host cell, *L. monocytogenes* escapes from the vacuole and spreads from cell to cell using actin polymerization (69).

Four nanobodies (Nbs), namely R303, R326, R330, and R419, exhibit strong binding to the LRR domain of InlB with nanomolar affinity (74, 75). This LRR domain is crucial for the interaction of InlB with c-Met. These Nbs can inhibit *in vitro* bacterial endocytosis and protect the cells from *Listeria* invasion (76).

Since preventing *L. monocytogenes* infection is the most effective approach, these Nbs in dimeric form or grafted on the Fc domain of human IgG should be tested *in vivo* to validate their therapeutic ability. Additionally, considering neutralizing Nbs targeted against other virulence factors like InlA is a significant expansion in the fight against listeriosis. Diversifying the arsenal of therapeutic agents to target multiple virulence factors can potentially enhance the efficacy of prevention and treatment strategies against *L. monocytogenes* infections. Further research and testing in this direction are vital for advancing our ability to effectively combat this pathogen.

### 2.3 Nanobodies to reduce *Campylobacter jejuni* loads in infected chickens

*Campylobacter jejuni* is a well-known foodborne pathogen responsible for human infections, with broilers identified as the primary reservoir. Reducing *Campylobacter* levels in the broiler caeca by at least 2 logs could significantly decrease the number of human infections by as much as 3 logs. In the past, antibiotics were added to the animal feed, resulting in a very dangerous increased antibiotic resistance (77, 78). To combat antibiotic resistance in *C. jejuni* strains, several other approaches were tested to prevent colonization of broilers by *C. jejuni*. These included the use of fatty acids, bioactive plant additives, probiotics, bacteriophages, bacteriocins and vaccination of young chickens with heat-killed *C. jejuni* bacteria. Unfortunately, none of these approaches lead to the desired *in vivo* effect (79–85).

In ovo immunization of embryos with a bacterin or a subunit vaccine were inoculated with C. jejuni at 19 days post-hatch. Quantification of *C. jejuni* in the broilers’ cecal content showed that the in-ovo-vaccinated birds were not protected against *C. jejuni* infection (86). However, a promising avenue emerged through passive immunization using anti-*Campylobacter* maternal IgY antibodies obtained from eggs of *C. jejuni*-immunized hens were shown to reduce the *C. jejuni* load in infected chickens (87).

Since passive immunization was promising as a therapeutic treatment (88, 89), Nbs against the purified recombinant flagellin were isolated and shown to reduce the motility of *C. jejuni* and the colonization in chickens (90). Alternatively anti-*C. jejuni* Nbs were isolated from a llama immunized with heat-killed *C. jejuni* (91). Among them, six Nbs targeted the major outer membrane protein (MOMP) of *C. jejuni* and exhibited a broad host range recognizing *C. jejuni* and *C. coli* strains isolated from chickens and humans. In addition to those, Nbs directed against the *C. jejuni* flagellin were also obtained from the same Nb library (92). These anti-MOMP and anti-flagellin Nbs were fused to the Fc domain of chicken IgA and expressed in *Arabidopsis thaliana*. The resulting plant-produced recombinant anti-MOMP Nb-IgA have the ability to bind to purified MOMP and effectively agglutinate *C. jejuni* strains, showing promise for targeting this pathogen. Furthermore, the anti-flagellin Nb-IgA plantibodies not only recognize the flagella of *C. jejuni* but also significantly reduce the motility of these bacteria (92). These findings underscore the potential of plant-based expression systems in generating functional antibodies to control bacterial pathogens like *C. jejuni.*

### 2.4 Treatment of *Bacillus anthracis* infections

Anthrax is an ancient zoonotic disease that primarily infects herbivores and occasionally leads to human infections (93). Its etiological agent is *B. anthracis*, a Gram-positive, aerobic, spore-forming, nonmotile, rod-shaped bacterium (93). Humans’ exposure to anthrax can occur through contact with infected animals and their derivatives, by direct contact with spores in the environment or by voluntary release of spores in case of a bioterrorist attack (94, 95). Once spores enter the host, they germinate into vegetative cells and reproduce within the host, releasing toxins that lead to acute septicemia and death (96).

Anthrax treatment typically involves antibiotics like penicillin, ciprofloxacin, and doxycycline, with the choice dependent on factors like infection site, time since exposure, and disease severity, including systemic signs of infection (93, 97–101). In cases of systemic or inhalational anthrax, the American Center for Diseases Control (CDC) recommends additional antitoxin treatment (102) in conjunction with intravenous antibiotics (98). It is important to note that these antitoxins are available solely in the USA.

Effective control of anthrax in humans hinges on managing the disease in animals. Spore vaccines have been a cornerstone of veterinary services in many countries since their large-scale use was first explored in the 1940s (103, 104). However, this vaccine, effective in many ways, raises concerns due to significant drawbacks, including residual virulence, batch variations, and the risk of environmental contamination (104–106). In humans, pre-exposure vaccination is provided by an acellular vaccine (anthrax vaccine adsorbed or AVA) (107) that contains anthrax toxin elements and results in protective immunity after three to six doses. Access to vaccination in the USA, Canada and several European countries is recommended only to people between 18 and 65 years old and limited to the ones at high risk of anthrax exposure (laboratory workers, veterinarians, military personnel, etc.) (107). In July 2023, the American Food Drug Administration (FDA) approved a novel human vaccine known as CYFENDUS™ (Emergent BioSolutions) for post-exposure prophylaxis, administered together with recommended antibacterial drugs following suspected or confirmed exposure to *B. anthracis*. This vaccine combines AVA with an additional adjuvant that has been shown to induce protective levels of immune response after just two doses administered over a 14-day period. This improved characteristic of AVA is crucial when facing a large-scale public health emergency involving anthrax.

Thus, in a scenario where low-income countries urgently need efficient anthrax drugs due to the constant risk of exposure, there is a call for efficient, affordable, and easily storable drugs to combat anthrax.

Anthrax disease is induced by three proteins: the protective antigen (PA), the lethal (LF) and edema (EF) factors, with PA functioning as an entryway that allows the translocation and activity of LF and EF toxins in the host cytosol (108). Antibodies targeting PA have been demonstrated to provide protection against the disease (109–111). In 2015, Moayeri and colleagues, upon alpaca immunization, identified two classes of Nbs with PA-binding capabilities, exhibiting anthrax toxin-neutralizing activity in macrophage toxicity assays (112). Remarkably, the authors reported enhanced neutralizing potency in cell assays and significantly improved efficacy in protection from anthrax toxins in a mice infection model when linking two Nbs targeting different neutralizing epitopes into a heterodimeric Nb-based neutralizing agent instead of using individual Nbs. A subsequent paper from the same research group identified a set of Nbs against the EF and LF components, demonstrating their therapeutic effectiveness in a spore model of anthrax infection in mice. This discovery opens a new strategy to treat anthrax by combining these EF/LF-neutralizing Nbs with anti-PA Nbs (113). One of the novel strategies for treating anthrax is to target the surface layer proteins (SLPs) of *B. anthracis.* SLPs self-assemble in Surface layers, or S-layers, intriguing two-dimensional protein arrays commonly observed on the surfaces of bacteria and archaea (114–116). *B. anthracis*, as part of its immune evasion strategy, possesses a complex and dynamic cell envelope composition (117) that includes switchable S-layers (118). In 2019, Fioravanti and co-workers made a significant breakthrough by immunizing llamas with the SLP Sap and identifying the first Nb presenting antimicrobial activity against *B. anthracis* (119). The identified Nb^AF692^ not only prevents Sap assembly but is also able to depolymerize Sap S-layers *in vitro*. Interestingly, although sera of Sap-immunized mice or llamas inhibited *de novo* Sap S-layer assembly, the S-layer depolymerizing activity was unique to Nbs, highlighting the unique steric properties of this single-domain antibody format. *In vivo*, the Nbs-mediated disruption of the Sap S-layer results in severe morphological defects (shriveled and collapsed cells) and attenuation of bacterial growth. Remarkably, the subcutaneous delivery of this Nb clears *B. anthracis* infection and prevents lethality in a mouse model of anthrax disease (119). This again demonstrates the therapeutic potential of Nbs in treating bacterial pathogens.

## 3 Nanobodies as therapeutics against viruses

### 3.1 Nbs against respiratory viruses

#### 3.1.1 Influenza virus

Seasonal flu is a recurring threat responsible for a significant number of human fatalities worldwide. This acute respiratory infection is caused by influenza viruses that circulate globally. The primary culprits behind human influenza infections or mortality are influenza A (IAV) and B (IBV) viruses (120). The first line of defense is vaccination with vaccines carrying IAV subtypes H1N1 and H3N2 and one or two IBV subtypes. Because of the high mutation rate, annual vaccination is needed to provide protection against the circulating mutant (120). In addition to the vaccination strategy, antiviral drugs targeting both seasonal and pandemic influenza strains are complementing the vaccination strategy. Nevertheless, it is worth acknowledging that both vaccination and antiviral drugs have their limitations, and there is a growing concern about the development of antiviral drug resistance (120). Furthermore, highly pathogenic, zoonotic avian influenza A viruses of the H5N1, H7N1, and H7N7 subtypes can cross the species barrier between domesticated birds and humans (121). Given these ongoing challenges posed by influenza virus infection, new approaches to tackle influenza virus infection are required. One of these approaches consists of the isolation of neutralizing Nbs targeting the two principal envelope proteins of the influenza virus, namely the hemagglutinin (HA) and the neuraminidase (NA). These two proteins are highly variable in terms of antigenic properties and are key to classifying the influenza A virus into diverse subtypes (for example human subtypes H1N1, H3N2, H2N2) (120). The HA plays a pivotal role by recognizing and binding to the sialic receptor on the surface of host cells, and it is responsible for virus entry. Conversely, NA is involved in the release of newly produced viral particles from the infected host cells (120).

Nbs with potent antiviral activity against influenza A viruses were isolated by several groups (122, 123). HA-specific H5N1- and H5N2-neutralizing Nbs were reported (120, 124). This is a very nice example of using Nb fusion to the Fc domain of human IgG1 for passive immunization against influenza.

Four broadly neutralizing Nbs (SD36 and SD38 against HA of influenza A and SD83 and SD84 against HA of influenza B) were isolated (125).To increase the potency and breadth, these four Nbs were fused genetically to create MD2407 (SD38–SD36–SD83–SD84) and MD3606 (MD2407 fused to human IgG1-Fc). These multidomain fusions neutralized all A (H1-H12, H14) and B viruses tested except for one avian H12 virus and are performing better than the human monoclonal antibody CR9114 (126). The MD3606 is neutralizing *in vitro* more influenza A and B viruses than the individual Nbs (SD36, SD38, SD83 and SD84) or the broadly neutralizing antibody CR9114 (125). Also, prophylactic efficacy studies have shown that MD3606 is more efficient in protecting BALB/c mice than CR9114.

In a more recent study, Nbs against the highly conserved stem domain (SD) of HA were isolated using a stabilized trimer (127). Among 66 Nbs, two Nbs with high titers in ELISA and high-affinity binding in surface plasmon resonance were tested *in vivo* in mice. Both Nbs showed complete neutralization of the tested H1N1 and H5N2 influenza viruses and complete protection of mice challenged with influenza virus (127).

Bioinformatic analysis identified two universally conserved epitopes within NA of all nine influenza IAV subtypes (128). One of these epitopes is in proximity to the NA enzymatic site, whereas the other is located near the NA N-terminus, which forms the cytoplasmic tail. Nbs generated against these epitopes after peptide immunization represent valuable candidates for targeting these critical regions of the NA protein across a range of IAV subtypes (128). Additionally, NA-binding Nbs targeting the zoonotic highly pathogenic avian influenza virus subtype H5N1 were successfully isolated and characterized (121). Among these anti-NA Nbs, some exhibited potent NA-inhibitory activity and *in vitro* and *in vivo* antiviral activities. These Nbs were produced as bivalent tandem formats in *E. coli* or after fusing the anti-NA Nbs to a mouse IgG2a Fc domain expressed in seeds of transgenic *Arabidopsis* plants (121). The tandem and IgG2a formats were tested *in vitro* for antiviral activities against H5N1 clade 1 (oseltamivir-sensitive and -resistant strains) and compared with those against clade 2 viruses. The bivalent constructs showed a 30- to 240-fold higher antiviral potency than that of their monovalent counterparts. In addition, prophylactic treatment with the bivalent or Fc-fused constructs protected mice against a potentially lethal infection with influenza virus H5N1, including an oseltamivir-resistant H5N1 variant (121). Moreover, a nanobody targeting the influenza virus M2 protein, capable of inhibiting the replication of amantadine-sensitive and -resistant viruses, was obtained. This nanobody proved effective in protecting mice against lethal influenza virus challenges (129). These breakthroughs demonstrate the potential of nanobodies as effective tools in the treatment of influenza infections, offering new approaches to tackle this persistent health threat.

#### 3.1.2 Respiratory syncytial virus

Respiratory syncytial virus (RSV) is a widespread respiratory pathogen responsible for annual epidemics, with a significant impact on vulnerable populations, including children, the elderly and individuals with weakened immune systems. This virus poses a substantial public health threat, particularly in developing countries, where it can lead to high mortality rates (130). Despite its impact, effective therapeutic options for RSV have remained elusive (131). Ribavirin, a broad-spectrum antiviral agent, exhibits limited efficacy against RSV. While the FDA has approved since 1986 the aerosolized formulation of ribavirin for use in hospitalized high-risk infants and young children, its use in adults remains unapproved (132, 133). However, recent studies have shown that ribavirin improves the survival of immunocompromised patients who have contracted RSV (134, 135). Efforts to advance RSV therapeutics, encompassing vaccines, extended-duration of mAbs and antiviral drugs, have been making rapid strides in recent years (136). In the landscape of monoclonal antibodies (mAbs), Palivizumab, a humanized mAb targeting the RSV fusion protein (F-protein), serves as the primary prophylaxis for preventing RSV disease in infants, significantly reducing RSV-related hospitalizations compared to placebo (137, 138). However, Palivizumab is unavailable in certain countries like China and poses a financial burden on low- and middle-income families (139). Consequently, alternative mAbs such as nirsevimab, engineered for longer half-life and easier delivery, have been developed and could provide potential alternatives by protecting infants throughout an entire RSV season with a single dose (140, 141).

A noteworthy milestone occurred in May 2023, when the FDA granted approval for the first RSV vaccine, marking a significant breakthrough that holds the promise of improving lives (142). Also, some novel antiviral candidates, like presatovir and lumicitabine, are promising in adult human challenge models but show challenges in obtaining a similar efficacy in target populations (143–145). In light of the ongoing need for innovative approaches to combat RSV, Nbs have emerged as potential candidates in the fight against this virus. The VIB-UGent lab led by Professor Saelens, along with collaborators, made significant contributions to the identification of Nanobodies (Nbs) against RSV. In a 2011 paper, Schepens et al. identified Nbs targeting the RSV protein F, which neutralized RSV by inhibiting fusion while sparing viral attachment. Intranasal administration of bivalent RSV F-specific Nbs protected mice from infection and associated pulmonary inflammation by reducing viral replication and lung inflammation (146). Subsequently, in 2017, another study from the same lab described llama-derived Nbs with potent RSV-neutralizing activity, targeting the prefusion RSV F protein and preventing viral replication and lung inflammation in RSV-challenged mice by blocking it in that state (147). These Nbs hold promise as therapeutic molecules for RSV treatment, although clinical trials are needed for validation. ALX-0171, a trivalent Nb targeting the RSV fusion protein to inhibit viral entry into host cells (**Figure 1C**), has successfully been developed as a nebulized solution for direct delivery to the site of infection in the lower respiratory tract by Ablynx (Sanofi). Promising results were obtained in a lamb model infected with a human RSV strain even if the therapy was given three days post infection (148). A preparatory multicenter study in young children with RSV lower respiratory tract infections showed rapid viral reduction in nasal RSV viral titers without safety concerns (EudraCT 2014–002841–23). To further assess the safety and antiviral activity of nebulized Nb in young children hospitalized with RSV lower respiratory tract infection, Cunningham and colleagues performed a phase 2b clinical trial. Unfortunately, the trial revealed that the observed decline in RSV viral load promoted in nasal mid-turbinate swabs by ALX-0171 treatment was not associated with a corresponding clinical improvement earlier observed in the lamb model (149). Following this study, no further development of ALX-0171 was planned.

#### 3.1.3 Severe acute respiratory syndrome coronavirus 2

In December 2019, an outbreak of pneumonia of unknown origin was identified in Wuhan, China (150, 151). On the 12^th^ of January 2020, Chinese authorities shared the sequence of a novel coronavirus termed severe acute respiratory syndrome coronavirus 2 (SARS-CoV-2) isolated from some clustered cases (152). SARS-CoV-2 is an enveloped positive-sense, single-stranded RNA (ssRNA) virus of the Betacoronavirus genus included in the Coronaviridae family (153). The virus possesses a trimeric spike (S) protein that decorates its surface. After binding the spike proteins to the host angiotensin-converting enzyme 2 (ACE2) receptor, the virus enters the host cell by fusing its envelope lipid bilayer with the target cell membrane (154).

Globally, on the 27^th^ of September 2023, there have been more than 770 million confirmed cases of COVID-19, including nearly 7 million deaths reported by the WHO. Thus, the SARS-CoV-2 virus has had a profound impact on global health. In response to this unprecedented global pandemic, massive efforts have been made worldwide to develop effective therapeutics aimed at saving countless lives. The currently most effective and FDA- and European Medicine Agency (EMA)- approved COVID-19 vaccines are the Pfizer-BioNTech and Moderna mRNA vaccines. Both vaccines encode the viral spike (S) glycoprotein (GP) of SARS-CoV-2. However, the high mutation rate of the spike protein results in the worrying emergence of several COVID-19 variants (155) that can evade host immunity (developed post-infection or vaccination), leading thus to new infection waves (156). Therefore, although these mRNA vaccines are very effective, not all vaccinated persons will be protected. Also, a significant number of people have refused vaccination. In the quest to find novel and more efficient therapeutic strategies to fight COVID, Nbs present several distinct advantages over traditional mAbs when it comes to combating SARS-CoV-2. Because of their peculiar characteristics, a more potent neutralization is observed in the case of Nbs compared to conventional mAbs against COVID-19. One significant advantage of Nbs is their capability to access cryptic epitopes that are conserved across different variants of SARS-CoV-2. This means that Nbs, thanks to their small size, can target hidden or less accessible regions on the viral surface, making them effective against a broader range of strains, including emerging variants of concern. Furthermore, Nbs are highly amenable to engineering, enabling the creation of modular and multimeric designs. This flexibility in design makes Nbs versatile candidates for developing broad-spectrum therapeutics that can adapt to new SARS-CoV-2 variants as they arise. In addition to that, their use in the context of a respiratory infection is a particularly attractive application, because they are very stable proteins and can be nebulized and administered at the site of infection (157{Esparza, 2022 #720, 158){Van Heeke, 2017 #678} (159).

Numerous SARS-CoV-2-Nbs targeting different epitopes have been identified using various strategies of selection and production. An overview of the neutralizing SARS-CoV-2-Nbs that were isolated by multiple research groups and their characteristics is described in a recent review article (160). Because all these Nbs were obtained upon S protein immunization, most neutralizing Nbs recognize and bind the receptor binding domain (RBD) present on the S protein. This RBD is present in two conformations: the “up” (accessible to the ACE2 receptor) and “down” (ACE2-inaccessible) conformation. Often these Nbs recognize and bind both conformations, impeding the binding of the RBDs to the ACE receptors, thus preventing the fusion between the virus and the host cell. Below, we will discuss some of these Nbs that have shown impressive neutralization *in vitro* as well as *in vivo*.

Ty1 is the first Nb isolated from an alpaca immunized with the S protein and found to be efficient in neutralizing the infection of SARS-CoV-2 *in vitro* (161). This Nb specifically targets the RBD in both its conformations, impeding the binding to the ACE receptor. CryoEM showed that the target epitope of Ty1 is usually shielded from conventional antibodies by glycans (162, 163), especially when the RBD is in the down conformation. Ty1, thanks to its specific format, can reach its epitope, which is usually masked by glycan at position N165 (161). The authors showed that the fusion of Ty1 to the Fc domain of human IgG1 enhances the neutralizing effect of Ty1, making it an even more potent COVID-neutralizing agent.

Two related Nbs, H11-D4 and H11-H4, bind the RBD with high affinity and block its interaction with the ACE2 receptor (164). Single-particle cryoEM and X-ray crystallography revealed that both Nbs bind the same epitope in all RBDs of the S protein trimer, which partly overlaps with the ACE2 binding surface, effectively obstructing the interaction between the RBDs and the ACE2 receptor. To increase their *in vivo* half-life and enhance avidity (165), the H11-D4 and H11-H4 Nbs were fused to the Fc domain of human IgG1 to produce a homodimer chimeric protein capable of bivalently binding the ACE2 receptor (**Figure 1C**). In an *in vitro* infection model, Nb-Fc fusions showed promising therapeutic neutralizing activities against SARS-CoV-2 and additive neutralization when tested together with the SARS-CoV-1/2 antibody CR3022 (154, 164, 166). Immunizing four camels with the SARS‐CoV‐2 spike RBD, Gai and colleagues (2021) identified Nb11–59, which also prevents RDB-ACE2 complex formation but recognizes the RBD of eight variants of SARS‐CoV‐2. This Nb showed *in vitro* a potent neutralizing efficiency near 50% against authentic SARS‐CoV‐2 and its variants. This Nb was humanized (HuNb11‐59)F for potential future clinical application (167, 168). HuNb11‐59 presents high stability between 4 and 40°C over two weeks. Also, there was no impact on protein stability after nebulization and no degradation upon freezing and thawing cycles. In addition to its high stability, the authors proved that HuNb11‐59 could be produced in large quantities in *Pichia pastoris* by fermentation with 20 g/L titer and 99.36% purity. These unique characteristics make Hu-Nb11‐59 a promising prophylactic and therapeutic molecule against COVID‐19 by direct inhalation (169).

In an effort to expedite the discovery of novel and more efficient therapies against COVID-19, researchers also employed a rapid approach for the isolation and characterization of Nbs. These synthetic single-domain antibodies, known as “sybodies” (Sb) (170), were obtained from available synthetic libraries (171). Thanks to this approach, Sb23 Nb, displaying a high affinity and neutralizing activity, was identified (172). Structural characterization of the Sb23-RBS complex revealed that Sb23 binds the RBD in both its “up” and “down” conformation and thereby effectively blocks competitively the RDB-ACE2 interaction (172). The first COVID-19-neutralizing, monomeric-Nb (non-Fc-fused Nb) was identified in 2021 (173). This camelid single-domain antibody Nb, K-874A, can disrupt the fusion of the viral membrane to the host’s cell membrane by preventing the S protein priming by the type II transmembrane serine protease TMPRSS2 (173, 174). Cryo-electron microscopy revealed that K-874A binds between the RBD and N-terminal domain of the virus S protein. In an *in vitro* infection model, K-874A shows excellent neutralizing ability in VeroE6/TMPRSS2 cells and human alveolar-derived cells. The monomeric-Nb presents an impressive S protein-binding affinity in nanomolar ranges when compared to the Fc-fused ones that we just described (173). *In vivo*, in a Syrian hamster model of infection, introducing K-874A through the nose decreased severe COVID-19 symptoms and limited infection signs in the animal’s lungs. In addition, K-874A subministration did not result in a massive cytokine storm, a life-threatening condition generally occurring after SARS-CoV-2 infection (173). Such evidence makes K-874A an excellent drug candidate to fight COVID-19.

We are still living in a time where the SARS-CoV-2 virus continues to circulate, and its high mutation rate often leads to mutations in epitopes targeted by neutralizing antibodies and nanobodies. Consequently, this can compromise the efficacy of these potential therapeutics, leading to diminished or even lost binding and neutralization capabilities. However, a combination of neutralizing nanobodies that target diverse critical sites on the SARS-CoV-2 virus, particularly the cryptic ones, could potentially offer prolonged efficacy in treating individuals infected with emerging virus variants.

### 3.2 Ebola virus

Ebola virus disease (EVD) is an exceedingly lethal illness that primarily affects both humans and nonhuman primates (175). EVD arises from an infection caused by a virus belonging to the Filoviridae family and the Ebolavirus genus (176). There are five identified Ebola virus species, four of which are known to cause disease in humans. The disease was first identified in 1976 by Dr. Peter Piot while investigating an alleged yellow fever case in the Democratic Republic of Congo (177). Sporadic outbreaks of Ebola disease predominantly take place in sub-Saharan Africa, gravely impacting the populations of these regions. Since its discovery, Ebola has posed complex diagnostic challenges and emerged as a substantial global public health threat partly due to the presence of significant immigrant populations in areas vulnerable to the disease. The largest Ebola outbreak occurred between 2014 and 2015 and was declared over in 2016 by the World Health Organization reporting approximately 28,000 cases and over 11,000 fatalities (178). Typically, EVD outbreaks originate from a single case of probable zoonotic transmission, followed by subsequent human-to-human transmission via direct contact or contact with infected bodily fluids or contaminated fomites.

In recent years, a significant and commendable scientific effort has been undertaken to prevent Ebola from escalating into a global crisis. In 2019, the first Ebola vaccine was approved by the FDA, and in 2020, two additional treatments have been approved for managing EVD caused by the Zaire Ebola virus species in both adults and children. The ERVEBO^®^ vaccine is a replication-competent, live, attenuated, recombinant vesicular stomatitis virus (rVSV) vaccine that expresses the EBOV GP antigen to stimulate an immune response. Because the GP is the sole surface protein of the EBOV virion and mediates attachment, fusion, and entry of target cells, this protein serves as an attractive immunogen, being readily recognized by the immune system and being the main target of the neutralizing antibody response (179). Currently, ERVEBO^®^ is the only vaccine with proven clinical efficacy and FDA and EMA approval. In theory, vaccination offers an ideal approach to combat EVD, but significant challenges impede the feasibility of this strategy. The necessity for an ultra-cold chain for long-term vaccine storage poses a substantial financial and logistical hurdle, especially in African countries. Additionally, limited vaccine acceptance within affected regions represents an obstacle to achieving the vaccination rates required to attain herd immunity against EVD. Consequently, achieving the necessary level of vaccination coverage remains a daunting challenge (180). Moreover, the current ERVEBO^®^ vaccine does not protect other Ebola virus species. Thus, in this context, it is essential to possess drugs that allow the treatment of EVD. In 2020, two anti-EBOV drugs were approved by the FDA: Inmazeb™ (181), a combination of three mAbs, and Ebanga™, a single mAb (182). These mAbs bind to the surface GP of the Ebola virus, preventing its entrance into host cells (182). Both treatments were evaluated during the 2018–2020 Ebola outbreak in the Democratic Republic of the Congo (183). Overall survival was much higher for patients receiving either of the two treatments. Neither Inmazeb™ nor Ebanga™ have been evaluated for efficacy against species other than the *Zaire Ebola virus*, leaving us with a need for novel drugs.

Additionally, there has been a growing interest in using Nbs to treat EVD. In 2021, Esmagambetov and co-workers, immunizing alpaca with a recombinant human adenovirus 5 expressing EBOV GP (Ad5-GP), obtained a promising Nb specifically binding the EBOV GP (184). The Nb, aEv6, showed a high affinity constant for its GP target as well as virus-neutralizing activity against the recombinant vesicular stomatitis virus pseudo-typed with the EBOV GP (rVSV-GP). To improve its pharmacokinetic and immunologic properties, the Nb was fused with the human IgG1 Fc fragment (**Figure 1C**). Such modification increased the lifespan of aEv6–Fc in the blood of non-human primates for up to 7 days instead of the several hours of the classical Nb (18, 185–187). *In vitro*, aEv6–Fc had specific binding activity and affinity like that of the Ebanga™ single mAb (MAb114) but a stronger virus-neutralizing activity than both the MAb114 and the unmodified aEv6 lacking the Fc fragment. In the light of such results, aEv6–Fc was then tested in a lethal model of murine rVSV-GP infection showing complete protection of mice when either pre-incubated with aEv6–Fc alone or mixed with the virus prior to infection. A 30% protection was observed when aEv6–Fc was administered no later than 2 h after infection with the virus (184). Although these findings indicate the need for improved protection and a longer timeframe between infection and administration of aEv6–Fc for real-life applications, they demonstrate the promising potential of aEv6-Fc as a protective agent for both prevention and treatment immediately after suspected contact with EBOV.

### 3.3 Human immunodeficiency virus

Human immunodeficiency virus (HIV) continues to be a significant global public health challenge. Until now, HIV has claimed between 32.9 to 51.3 million lives, with ongoing transmissions still occurring in all countries worldwide. In 2022, approximately 630,000 people died from HIV-related causes. Additionally, an estimated 39.0 million individuals are living with HIV, with the majority (25.6 million) residing in the WHO African Region. HIV is a retrovirus that is transmitted via body fluids and secretions that mainly infects clusters of differentiation 4-positive (CD4+) cells, with a strong preference for CD4+ T helper lymphocytes (188). To successfully invade the host cell, HIV requires, in addition to the CD4 receptor, also a coreceptor, that is either the C-C chemokine receptor type 5 (CCR5) or the C-X-C chemokine receptor type 4 (CXCR4). The HIV envelope protein (HIV Env) consists of two glycoproteins, gp120 and gp41, which mediate viral attachment and host membrane fusion, respectively (189). The fusion process is initiated by gp120 after binding to the host CD4 receptor and CXCR4/CCR5 coreceptor induces a conformational change in gp41, resulting in the fusion of the viral and host cell membranes (190, 191). Infection with HIV ultimately leads to host cell death and a consequent depletion of CD4+ T lymphocytes (192). Since CD4+ T lymphocytes play a vital role in regulating the adaptive immune system, their depletion significantly weakens the immune system. This weakening of the immune response is a hallmark of the acquired immune deficiency syndrome (AIDS) stage of the HIV infection (193) that compromises the body’s ability to fight off infections and diseases, making individuals with advanced HIV infection more susceptible to other infections and health complications associated with AIDS. The development of rapid diagnostics and effective antiretroviral therapy led worldwide to a large reduction in mortality and morbidity and to an expanding group of individuals requiring lifelong viral suppressive therapy. Although antiretroviral therapy (ART) can reduce plasma virus levels below detection limits (≤ 50 copies/ml), long-term suppression of HIV replication by ART cannot totally eliminate HIV (194, 195). The virus unfortunately persists in cellular reservoirs thanks to cryptic ongoing replication, viral latency and/or poor drug penetration (195–197) because HIV RNA returns to a measurable plasma level in less than two weeks when ART is interrupted (198). To date, no HIV vaccine or cure exists despite years of intense research efforts and a clear need of them. In this context, over the last years, a plethora of neutralizing HIV Nbs targeting HIV gp120 and gp41 have been identified and extensively reviewed (199). To increase the potency of HIV neutralization, anti-HIV Nbs have been modified to bivalent and trivalent Nbs recognizing the same or distinct epitopes on the HIV Env or fusing them to human Fc domains of IgG (199). In 2023, a novel and very promising Nbs-based curative therapy against HIV was developed (200). The authors successfully constructed a bispecific complement engager (BiCE) that comprises a Nb recruiting the complement-initiating protein C1q (201) fused to a single-chain variable fragment (scFV) of two broadly neutralizing antibodies, the bNAb 10–1074 or the 3BNC117 (202, 203) that target the HIV Env (**Figure 1C**). These two anti-HIV BiCEs can recognize the HIV Env and neutralize free virus in an *in vitro* virus neutralization assay. Furthermore, both anti-HIV BiCEs were reported to mediate *in vitro* complement activation by increasing C3 deposition on HIV Env-expressing Raji cells and consequently promote complement-dependent lysis of the latter (200). The results of anti-HIV BiCEs hold significant promise for a therapeutic strategy aimed at addressing HIV infection. The use of anti-HIV BiCEs is enhancing complement-mediated killing of HIV-infected cells, offering a potential solution to one of the major hurdles in curing HIV: the persistence of a latent HIV virus (204). In a prospective scenario, this approach could involve a combination of strategies to target HIV infection. Firstly, a treatment employing latency reversal agents could be used to activate latent HIV-infected cells. Following the activation of latent cells, the next crucial phase would be to boost the immune response (205). In this aspect, the use of anti-HIV BiCEs, which facilitate complement-mediated killing, could play a pivotal role (200). These bispecific antibodies would enhance the immune system’s ability to recognize and destroy the reactivated HIV-infected cells. This multi-pronged approach, involving both the activation of latent cells and the reinforcement of the immune response, holds a significant potential for advancing the pursuit of an HIV cure and represents a significant step forward in the quest to address the complex challenges of HIV infection and its latent reservoir.

### 3.4 Herpes simplex 2 virus

Herpes simplex virus 2 (HSV-2) ranks among the most prevalent sexually transmitted infections globally, infecting approximately 16% of individuals aged between 15 and 49 (206). While generally not life-threatening, HSV-2 can lead to severe complications, particularly in immunocompromised individuals and infants (207). Moreover, HSV-2 infection is linked to a significantly higher risk of contracting HIV (208, 209). Making prevention more challenging, a substantial portion of primary HSV-2 infections and reactivations go unnoticed, as they are subclinical, allowing asymptomatic individuals to unknowingly transmit the virus (210, 211). Vaccine strategies designed to prevent HSV-2 transmission have encountered limitations in terms of their broad effectiveness. Additionally, relying solely on condoms for protection is not always foolproof (212). In response to these challenges, there has been a focus on investigating alternative methods for preventing and treating HSV-2, among which Nbs have emerged as a promising avenue. Geoghegan and colleagues identified a Nb called R33 after immunizing llamas with HSV-2 GP D (213). Notably, R33 on its own, does not exhibit HSV-2 neutralization activity *in vitro*. However, when combined with the cytotoxic domain of *Pseudomonas aeruginosa* (**Figure 1C**), it resulted in an immunotoxin known as R33ExoA, demonstrating the ability to specifically and potently eliminate HSV-2-infected cells. Its 50% neutralizing dilution is measured at 6.7 nM, showcasing its potential as a highly effective therapeutic agent against HSV-2 infection (213). These findings suggest the potential clinical utility of R33ExoA for preventing HSV-2 transmission by eliminating virus-producing epithelial cells during viral reactivation. Furthermore, R33 may serve as a versatile platform for delivering other cytotoxic effectors to HSV-2-infected cells, indicating its broader therapeutic applicability beyond HSV-2 infection.

### 3.5 Human papilloma virus

Human papillomavirus (HPV) has been linked to nearly 5% of all cancer cases worldwide (214). HVP is a group of over 200 related viruses, with 15 of them being carcinogenic and classified as high-risk HPV (215). HVP is renowned as one of the most common sexually transmitted infections and progresses from asymptomatic infection to the development of warts at the site of infection or to more serious benign or malignant cancers. These cancers encompass gastrointestinal, cervical, urinary bladder, and head and neck cancers (216). Alarmingly, these diseases collectively afflict more than half a million individuals worldwide every year, contributing significantly to cancer-related mortality in developing countries (217–219).

HPV is a small and non-enveloped virus with double-stranded circular DNA (218, 220) with a life cycle that takes place in keratinocytes under differentiation (221). HPV enters its host cells via the viral L1 capsid protein (222) where it replicates. Keratinocytes are found in the epidermis of the oral cavity, esophagus, and squamous epithelium of the genitals. A traumatic event at the epithelium facilitates HPV entry into basal epithelial cells and maintains the viral episome in the infected cells (223). Three HPV proteins, the E5, E6 and E7 proteins, have been shown to act as the main determinants in the oncogenic properties of HPV (224–227). Together, they act to prolong the host keratinocytes’ proliferation, delaying their differentiation and providing a suitable environment for viral replication.

In 2006, a significant milestone in public health was reached: the FDA approved the first vaccine against HPV. HPV vaccines have since played a crucial role in safeguarding public health by reducing the prevalence of HPV-related diseases by vaccinating young adolescent girls in most countries (228). Since 2009, the vaccine has also been approved by the FDA for boys (229), and an increasing number of countries worldwide are making efforts to raise awareness among boys and men to get vaccinated, aiming to achieve maximal vaccine coverage in the population. Currently, there are six licensed HPV vaccines available, all composed of viral L1 capsid proteins produced by different HPV subtypes and proven to be highly effective in preventing precancerous cervical lesions resulting from these virus types.

Preventative measures like vaccines and regular screenings are essential in the fight against HPV. However, there is still a need for effective therapies to treat current infections and cancers that are still a major cause of morbidity and mortality, including cervical and head and neck cancers caused by HPV (230). Additionally, the high cost of vaccine production and storage, the duration of HPV vaccine efficacy and coverage of HPV types remain important issues that must be faced (231). In this context, the need for a therapy is evident, but to date, an approved therapy against HPV is not available (232). Prior research suggested that inhibition of E6 and/or E7 function inhibits the growth of HPV-positive cervical cancer cells (233–235). Two main approaches were used to prove that in *in vitro* models of HPV infection: E6 and E7 RNA interference by siRNA (235) and the use of antibodies or small peptides targeting the E7 oncoproteins (236). This latter approach has identified a small peptide targeting HPV16 E7 that can bind and degrade E7, inducing a G1-phase arrest and suppressing the proliferation of SiHa cells *in vitro* and inhibiting SiHa tumor growth in mice (233, 237). In this context, once again, Nbs represent promising molecules for the generation of new HPV diagnostics and therapeutics. In 2012, Minaeian and coworkers reported the identification of a Nb against the HPV16 major capsid protein L1 able to neutralize HPV infection in an *in vitro* model of infection (238). In 2019, Li and colleagues were able to identify a Nb against the HPV16 E7 oncoprotein, Nb2, that, if transfected in HPV16-positive cancer cells and used as intrabody (intracellular antibody), would inhibit the growth of these cells, enlightening the potential and promising application of intrabodies for the therapy of HPV16-associated disease (239). With the same intent, Nbs against the HPV16 E6 oncoprotein were identified to be used as intrabodies. The discovery of a Nb9 capable of binding to the endogenous HPV16 E6 protein within HPV16-positive CaSki and SiHa cells is a noteworthy development. When this Nb was introduced and overexpressed in HPV16-positive SiHa and CaSki cells, several significant outcomes were observed. Notably, the localization of HPV16 E6 to the nucleus was inhibited, preventing the inactivation of p53 and leading to an increase in apoptosis. Additionally, the inhibition of tumor growth was evident in a mouse xenograft model (240). These Nbs open a promising avenue for the treatment of HPV-related conditions. The ability to target and modulate the activity of HPV16 E6 through Nb9 and HPV16 E7 by Nb2 offers potential therapeutic benefits, particularly in the context of HPV-associated cancers. Further research and development in this direction may yield innovative approaches to manage and treat HPV infections and their associated health risks. Additionally, despite the significant advancements made in the field of intrabodies over the past years, more research needs to be conducted to overcome the biggest challenge in translating neoantigen-directed intrabodies to the tumor cells in the clinic, as the specific targeting of the intrabodies to the tumor cells in an *in vivo* context remains challenging (241).

### 3.6 Hepatitis C virus

Hepatitis C virus (HCV) is an enveloped virus carrying a positive-sense ssRNA genome. The virus is primarily transmitted via injection of drugs, blood transfusion of unscreened donors, sexually, unsafe medical equipment or needlestick injuries. HCV infections are causing liver diseases that can be acute or chronic (242). Chronic liver inflammation can progress to fatal cirrhosis and hepatocellular carcinoma. Today, no vaccine against HCV is available and prior to advancements in medication, hepatitis C treatment hinged primarily on a regimen involving interferon and ribavirin. Patients received weekly injections of pegylated interferon alfa (PEG-IFNα) alongside daily oral ribavirin intake, a guanosine analog, to moderate the clinical symptoms and to limit the viral load (243). This therapy, however, was not only lengthy and stringent but also produced severe adverse effects (244). Fortunately, a significant breakthrough occurred with the introduction of a new generation of direct-acting antiviral (DAA) medications. These include Elbasvir/Grazoprevir (Zepatier), Glecaprevir/Pibrentasvir (Mavyret), Sofosbuvir/Ledipasvir (Harvoni), and Sofosbuvir/Velpatasvir (Epclusa), which have demonstrated remarkable efficacy in curing the virus while causing minimal side effects (245–247). Unfortunately, the widespread adoption of DAAs for treating HCV infection in many countries, particularly in low- and middle-income countries, has been impeded by the prohibitive cost of these medications (248).

Thus, especially before 2014, in a quest to find alternative therapies with lower or no side effects and considering the imperative of affordability, several research groups isolated Nbs against HCV. These Nbs target the E2 envelope GP (249), the intracellular HCV proteins RNA-dependent RNA polymerase (RdRp) (250) and the virus’ helicase (251) and serine protease (252).

Four distinct Nbs were generated from an alpaca immunized with the HCV E2 GP (253). One of them, Nb D03, recognized an epitope on the E2 GP that overlaps with the epitopes of several broadly neutralizing human mAbs (253). Nb D03 neutralizes six HCV genotypes by hampering the interaction of the E2 GP with its host receptor CD81. In this way, this Nb efficiently inhibits the cell-to-cell transmission of HCV (253).

An alternative strategy is to target intracellular HCV proteins with Nbs. The first target for developing anti-Hepatitis Nbs was the HCV’s RdRp (250). Nanobodies inhibiting the RdRp *in vitro* were subsequently fused to a 16-amino-acid, cell-penetrating peptide, penetratin (254), to produce cell-penetrable Nbs (**Figure 1C**). After adding these transbodies to human hepatic Huh7 cells transfected with the RNA of the HCV strain JFH1, the cell-penetrable Nbs, unfortunately, did not wholly suppress replication of the HCV RNA genome (250). The advantage of these cell-penetrable Nbs is the cross-neutralization of RdRp of other heterologous HCV genotypes since all HCV genotypes are highly conserved. A second intracellular target is the HCV helicase protein. A Nb was identified to bind the domain 3 of the helicase, which is necessary for its activity (251). This Nb was also fused to penetratin and shown to reduce the amount of HCV RNA that was released into the cell culture fluid and inside Huh7 cells transfected with RNA of the HCV strain JFH1 (251). A third intracellular HCV protein is a serine protease essential for processing the viral polyprotein replication in cell cultures and chimpanzees (255). Therefore, this protease is an attractive target for developing novel anti-HCV therapies. Three Nbs against the recombinant protease were isolated and fused to the penetratin peptide (252). In transfected Huh7 cells with RNA of the HCV strain JFH1, one of these cell-penetrable Nbs inhibits the replication of the HCV slightly better than the combined PEG-IFNα and ribavirin treatment or treatment with the protease inhibitor telaprevir. These promising results obtained in cell lines urge us to evaluate these Nb constructs’ efficacy in animal HCV infection models.

### 3.7 Rotavirus

Rotavirus has a genome of 11 segmented double-stranded RNAs. Of the nine species of known rotaviruses, the rotavirus A (RVA) species mainly causes acute gastroenteritis in infants and young children worldwide (256, 257). Two proteins on the surface of the virus determine the serotype of RVA. The GP VP7 defines the G serotypes, and the protease-sensitive protein VP4 defines the virulence and the P serotypes (258). At least 36 G- and 51 P -types are known, but only a few combinations of G and P types infect humans (259, 260). VP4 must be cleaved by trypsin in the gut into VP5 and VP8 before the virus is infectious (261). The inner capsid protein is formed by the highly conserved VP6, which is very immunogenic (262).

Vaccines against RVA infections were shown to be safe and effective in children (263). The WHO recommended rotavirus vaccination to be included in all national immunization programs (264). A result of these vaccinations in countries implementing this WHO recommendation is a significant reduction in the incidence and severity of rotavirus infections. The hospitalizations due to rotavirus infection in young children in fact also dropped between 49% and 92%, depending on the country (265).

Even though vaccination is very successful in many developed and some developing countries, rotavirus infections still occur in young children. A possible alternative strategy to treat those infections is passive immunization. Passive protection was shown in suckling mice fed with classical mAbs against the heterotypic neutralization domain of VP7 and the VP8 domain of VP4 (266). Also, VP6-specific secretory IgA mAbs were shown to induce intracellular viral inactivation in BALB/c mice, although VP6 is not exposed on the surface of the rotavirus particles (267, 268). However, two research groups showed that Nbs directed against VP6 can neutralize a wide range of RVA strains *in vitro* (269, 270), suggesting that the conserved nature of this protein allows cross-targeting of RVA strains.

Twenty-three rotavirus-specific Nbs were obtained after immunization of a llama, of which eight could be produced in yeast and showed *in vitro* neutralization of the rotavirus (271). The four Nbs with the highest production yield were tested in mice, showing a dose-dependent neutralization of the rotavirus strain in mouse pups (271). In a follow-up study, two of these four Nbs (ARP1 and ARP3) were further tested and shown to neutralize a wide variety of rotavirus serotypes and genotypes *in vitro*, including genotypes mostly found in infantile diarrhea. These Nbs could also reduce the infection level in a mouse pup model (272). Consequently, the ARP1 Nb was also evaluated in a clinical trial in infants with rotavirus infection in Bangladesh, showing that oral administration of ARP1 Nbs produced in yeast was safe and effective in reducing diarrhea in infants with severe rotavirus-associated diarrhea (273). In another study, it was shown that oral administration of anti-VP6 Nb has a prophylactic effect against RVA-associated diarrhea. Furthermore, these anti-VP6 Nbs are safe and active against diarrhea (110).

## 4 Discussion

Passive immunization through natural means is exemplified by transferring maternal IgG antibodies to the fetus via the placenta in humans and monkeys. Conversely, ruminants, horses, and pigs do not experience prenatal IgG transfer. Instead, these animals rely on neonates ingesting colostrum, which is absorbed into their bloodstream through the gastrointestinal tract within the first 24 hours after birth. On the other hand, mice, rats, and dogs receive maternal IgGs both *in utero* and through the gastrointestinal tract. Furthermore, immune serum from convalescent humans or animals, typically obtained from horses, has historically been used to treat patients. More recently, monoclonal IgG antibodies have expanded the range of applications for curing microbial diseases. These monoclonal antibodies (mAbs) offer enhanced efficiency and specificity, resulting in fewer adverse effects than whole serum treatments. The discovery of heavy-chain-only antibodies in camelids and the subsequent development of single-domain antibodies, known as Nanobodies (Nbs), have introduced numerous innovative strategies and expanded possibilities in the field of passive immunization.

Infectious diseases continue to pose a significant global threat to human health. The rapid spread of diseases like COVID-19 has shown the world the urgent need for improved prevention and treatment methods. Our review reveals that Nbs offer a promising alternative for combating bacterial and viral outbreaks. The majority of the described Nbs prevent the entry of pathogens into host cells by targeting bacterial or viral proteins that are exposed on the pathogen’s surface and are used to bind to the host’s receptor. In just one case, a Nb was found to act as an antimicrobial agent, targeting *B. anthracis* by disrupting its outermost cell surface component, known as the S-layer (119). This discovery marks the first example of a Nb exhibiting antimicrobial properties and provides initial evidence that the disruption of S-layer integrity holds therapeutic promise for S-layer carrying pathogens. The broad spectrum of Nb applications reviewed here underscores their exceptional versatility in combatting infectious diseases. In 2023, a ground-breaking study on Nbs targeting HIV introduced a novel and highly promising Nb-based curative therapy for HIV (200). This innovative approach, involving a bispecific complement engager (BiCE) that combines a Nb recruiting the complement-initiating protein C1q with single-chain variable fragments of broadly neutralizing antibodies targeting the HIV-1 envelope protein (**Figure 1C**), not only shows great potential for addressing HIV infection therapeutically but also paves the way for combatting other infectious diseases through complement-mediated killing of infected cells.

As outlined in our review, Nbs possess exceptional qualities such as profound tissue penetration, high affinity, structural adaptability, and cost-effective expression systems. These attributes open innovative avenues for preventing and treating infectious diseases. The potential applications of Nbs are extensive, and recent clinical and experimental data suggest that the development of multimeric and functionalized molecules using Nbs will play a substantial role in future diagnostic and therapeutic tools, especially in the context of infectious diseases. Nonetheless, there is still much to uncover and comprehend before translating Nb research into practical applications. Achieving this goal will require collaborative efforts from future researchers, promising a novel approach to treating a wide range of infectious diseases, ultimately enhancing human life and health.

## Author contributions

HD: Conceptualization, Writing – original draft, Writing – review & editing. AF: Conceptualization, Writing – original draft, Writing – review & editing, Funding acquisition.
